# Evaluation of Hedgehog Pathway Inhibitors as a Therapeutic Option for Uterine Leiomyosarcoma Using the Xenograft Model

**DOI:** 10.1007/s43032-021-00731-y

**Published:** 2021-10-12

**Authors:** Natalia Garcia, Mara Ulin, Mohamed Ali, Ayman Al-Hendy, Katia Candido Carvalho, Qiwei Yang

**Affiliations:** 1 Department of Surgery, University of Illinois at Chicago, Chicago, IL USA; 2 Laboratório de Ginecologia Estrutural e Molecular - LIM 58, Disciplina de Ginecologia, Hospital das Clinicas da Faculdade de Medicina da Universidade de Sao Paulo, HCFMUSP, Sao Paulo, Brazil; 3 Department of Pathology, University of Illinois at Chicago, Chicago, IL USA; 4 Clinical Pharmacy Department, Faculty of Pharmacy, Ain Shams University, Cairo, Egypt; 5 Department of Obstetrics and Gynecology, University of Chicago, Chicago, IL USA

**Keywords:** Uterine leiomyosarcoma, Hedgehog signaling, SMO inhibitor, GLI inhibitor

## Abstract

Uterine leiomyosarcoma (LMS) contributes to a significant proportion of uterine cancer deaths. It is a rare and high-risk gynecological cancer. LMS is challenging to the treatment due to the resistance of several therapies. The activation of the Hedgehog (HH) pathway has been reported in several types of female cancers. Uterine LMS presents an upregulation of the crucial HH signaling pathway members such as SMO and GLI1. Although targeting the HH pathway exhibited a potent inhibitory effect on the phenotype of uterine LMS in vitro, the effect of the HH inhibitors on LMS growth in vivo has not been identified. The present study aimed to assess the effect of Hedgehog pathway inhibitors (SMO-LDE225 and GLI-Gant61) as a therapeutic option in the xenograft model of uterine LMS. The results demonstrated that LDE225 treatment did not show any inhibitory effect on LMS tumor growth; however, treatment with GLI inhibitor (Gant61) induced a remarkable tumor regression with a significant decrease in Ki67 expression, compared to control (*p* < 0.01). Moreover, administration of Gant61 decreased the expression of GLI1, GLI target genes *BMP4* and *c-MYC* (*p* < 0.05), indicating that the HH pathway is implicated in the LMS experimental model. In conclusion, our studies demonstrate for the first time that GLI inhibitor (Gant61), but not SMO inhibitor (LDE225), shows a potent inhibitory effect on LMS tumor growth and concomitantly suppresses the expression of GLI1- and GLI-targeted genes using the xenograft model of uterine LMS.

## Introduction

Uterine leiomyosarcoma (LMS) contributes to a significant proportion of uterine cancer deaths [1–4]. It is a rare and aggressive gynecological cancer, which accounts for 1% of all uterine malignancies. LMS is challenging to treatment exhibiting resistance to several therapies including FDA-approved drugs such as pazopanib and olaratumab [2, 5–8], evidenced by high rates of recurrence and progression [9, 10]. These characteristics emphasize the need for new therapeutic options for this tumor.

The Hedgehog (HH) pathway activation depends on the HH ligand (SHH, IHH, or DHH) [11]. In the absence of the ligand, the PTCH1 receptor blocks SMO activity. However, when the HH ligand binds to PTCH1, the SMO inhibition is relieved, triggering activation and nuclear translocation of the GLIs transcription factors to regulate the HH target genes. In the absence of the HH ligand, the negative regulator SUFU sequesters the GLI proteins in the cytoplasm [12–14].

The deregulation of the HH signaling pathway plays an important role in more than 30% of human cancers [15]. This deregulation of the HH pathway contributes to tumor initiation and progression [16–19]. The activation of the HH pathway has been described in several types of female cancer, including uterine LMS [20–27]. In LMS, the HH pathway was described for the first time by Garcia and collaborators [25], who showed that the protein expression of SMO and GLI1, the crucial members of the HH signaling pathway, was increased in formalin-fixed paraffin-embedded (FFPE) LMS’ patient samples, compared to uterine leiomyoma and myometrium [25].

Due to the important role of key HH components in cancer progression, targeting SMO and the GLIs have been demonstrated to be a useful strategy to block the HH signaling pathway activity and suppress the tumor progression [28–33]. SMO and GLI inhibitors have been shown to exert an anti-cancer activity in vitro and in vivo on different types of cancer [20, 26, 34–36]. Furthermore, several SMO inhibitors are approved by the FDA (GDC099, LDE225, and PF-04449913) and have been tested in clinical trials showing promising results in breast cancer [37], basal cell carcinoma [38, 39], medulloblastoma [40], and pancreatic cancer [41].

Recently, our in vitro study demonstrated that LDE225 (SMO) and Gant61 (GLI) inhibitors were capable of blocking the HH pathway signaling with a significant decrease in the LMS cell proliferation and migration with prominent apoptosis enhancement [26]. However, the effects of the HH inhibitors on LMS growth in vivo are unclear. The present study aimed to assess the effect of HH pathway inhibitors as a therapeutic option using the xenograft model of uterine LMS.

## Material and Methods

### Cell Culture and Reagents

The human uterine LMS cell line (SK-UT1, ATCC® HTB-114™) was purchased from the ATCC (Manassas, VA, USA), and it was cultured in the recommended media and growth condition. GLI inhibitor, Gant61, was purchased from Sigma Aldrich (St. Louis, MO, USA) and SMO inhibitor LDE225 from Selleck Chemical (Houston, TX, USA).

### Leiomyosarcoma Xenograft Tumors

Twenty-nine nu/nu nude mice were purchased from Charles River (Wilmington, MA, USA). The mice were handled according to the approved protocol (18–174) and all mice were maintained in a 12-h light/dark cycle and provided with water and standard diet ad libitum in a pathogen-free facility under climate control. 2 × 10^7^ of the human LMS cells were inoculated into the right flank of mice with 1:1 Matrigel (Corning, Corning NY, USA) and fetal bovine serum (FBS) according to the previous publications [42–46]. After the tumor development, the animals were randomized separately into three groups, SMO inhibitor LDE225 (*n* = 5), GLI inhibitor Gant61 (*n* = 6 × 2), and control (*n* = 6 × 2). Gli treatments were repeated compared to the vehicle control group to receive enough tissues for cellular and molecular analysis due to Gant61’s marked inhibition of tumor growth; 20 mg/kg of LDE225 [47], 20 mg/kg of Gant61 [20, 48, 49], or corn oil (vehicle) were administrated via oral gavage three times per week for 10 days to the Gant61 group and 21 days to the LDE225 group. After the treatment, the animals were sacrificed, and tumors were collected for histopathological and RNA and protein expression profile analysis.

### RNA Extraction and Gene Expression

The total RNA was isolated using TRIzol Reagent (Invitrogen, CA, USA). The concentration was determined using NanoDrop (Thermo Scientific, Waltham, MA). The High Capacity cDNA Transcription Kit (Thermo Scientific, Waltham, MA) was used to perform the reverse-transcribed to complementary DNA by one microgram of the total RNA. The real-time PCR was performed using the CFX96 PCR instrument using SYBR Green Supermix (Bio-Rad, Hercules, CA, USA). The results are presented as relative gene expression using CFX Maestro™. The primers for detecting the gene expression profile are listed in Table 1.

**Table 1:** qRT-PCR primers sequences

Gene/symbol	Forward sequence	Reverse sequence
*GLI1*	AGCCTTCAGCAATGCCAGTGAC	GTCAGGACCATGCACTGTCTTG
*GLI2*	CTGTGGGTTAGGGATGGACTG	GTAAAGTGGGTGGACGTTGCA
*GLI3*	GTGCTCCACTCGAACAGA	TCCAGGACTTTCATCCTCATTAGA
*SMO*	TGAAGGCTGCACGAATGAGG	CTTGGGGTTGTCTGTCCGAA
*BCL-2*	TGTGTGTGGAGAGCGTCAAC	GCCAGAGAAATCAAACAGAGG
*CCND1*	CTTCAAATGTGTGCAGAAGG	CTCGCACTTCTGTTCCTC
*P21*	CCCTTGTCCTTTCCCTTCAGTAC	GTGGGACAGGCACCTCAGA
*BMP4*	CGTAGCCCTAAGCATCACTCACA	GCGCCGGCAGTTCTTATTCT
*FoxM1*	GGGCGCACGGCGGAAGATGAA	CCACTCTTCCAAGGGAGGGCTC
*c-MYC*	AATGAAAAGGCCCCCAAGGTAGTTATCC	GTCGTTTCCGCAACAAGTCCTCTTC
*P27*	ATGTCAAACGTGCGAGTGTCT	TTACGTTTGACGTCTTCTGA
*VEGF*	CTACCTCCACCATGCCAAGT	GCAGTAGCTGCGCTGATAGA
*TP53*	TGTAGTGGATGGTGGTACAG	CGTGTGGAGTATTTGGATGAC
*B2M*	CAGCCCAAGATAGTTAAGTG	CCCTCCTAGAGCTACCTGT

### Morphological (H&E) and Immunohistochemistry (IHC) Assessment

The LMS tumors were fixed in 10% buffered formalin for 24 h, then embedded with paraffin and subjected to H&E and IHC staining by Research Histology and Tissue Imaging Core at the University of Illinois at Chicago. The antibodies used in this study are shown in Table 2. The IHC analysis was performed using a semi-quantitative score considering the percentage of labeled cells (0, negative; 1, < 10% of the cells; 2, 10–50% of the cells; 3, 50–75% of the cells; 4, > 75% of the cells) and the intensity of the immunostaining (0, no staining; 1, weak; 2, mild; 3, strong staining). The multiplication of both scores resulted in a final quotient ranging from 0 to 12 [25]. The slides were scanned using the Aperio image scope software (Aperio Technologies, Inc., Vista, VA, USA).

**Table 2:** Description and details of antibodies used in this study

Antibody	Manufacturer	Species, monoclonal, or polyclonal	Application and dilution	Catalog number
SMO	GeneTex	Rabbit, polyclonal	IHC, 1:400	GTX02530
GLI1	Sigma	Rabbit, polyclonal	IHC, 1:200	ABC217
KI67	Abcam	Rabbit, monoclonal	IHC, 1:200	ab16667

### Statistical Analysis

The data are expressed as the means ± standard error by using the GraphPad Prism 5 software. Statistical analyses were carried out using the parametric (Student’s *t*-test or analysis of variance ANOVA followed by Tukey post-test) or nonparametric distribution (Mann–Whitney test or Kruskal–Wallis followed by Dunns post-test). The significant difference was defined as *p* < 0.05.

## Results

### Inhibition of Hedgehog Pathway Promotes Tumor Regression in the Xenograft Model of Uterine LMS

**Fig. 1:**
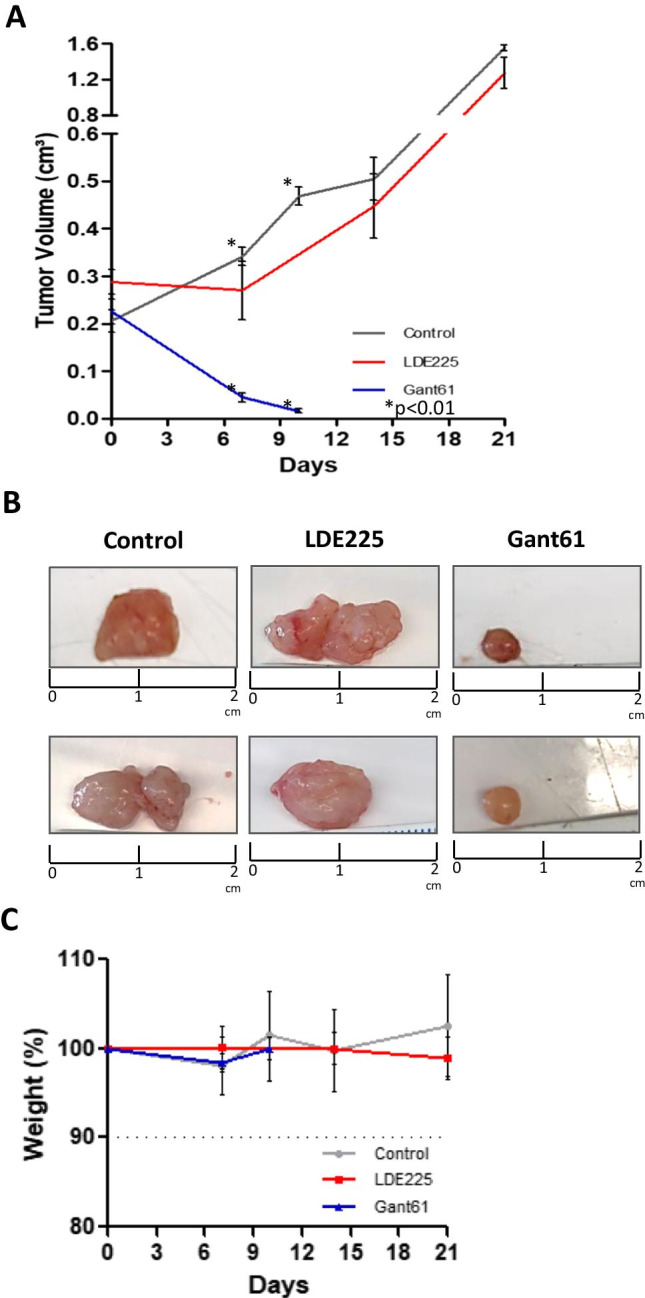
Response curve of LMS tumors treated with SMO (LDE225 20 mg/kg) or GLI inhibitors (Gant61 20 mg/kg). **A** The animals bearing tumors were administered with 20 mg/kg of LDE225 (SMO inhibitor) or Gant61 (GLI inhibitor), and the tumor growth was measured during the treatment. The relative tumor volume was calculated and plotted. **B** Pictures represented the tumor volume at the end of the treatment with SMO or GLI inhibitors compared to control. **C** Graph summarizing the percent body weight of mice during the treatment with SMO or GLI inhibitor

The HH inhibitors, SMO-LDE225 and GLI- Gant61, were selected based on our previous findings that these two HH inhibitors exhibited a significant inhibitory effect (decreasing cell proliferation, migration, and increasing apoptosis index) on LMS cells in vitro [26]. In this study, after the tumors developed, the animals were randomly separated into three groups (SMO inhibitor, GLI inhibitor, and control). SMO inhibitor group was treated for 21 days with 20 mg/kg of LDE225. In contrast to the in vitro inhibitory effect, the LDE225 treatment did not show any inhibitory effect on LMS tumor growth. In addition, no significant difference in tumor volume between the LDE225 treatment and the control group was observed (Fig. 1A, B). However, the animals treated with 20 mg/kg of Gant61, GLI inhibitor, for 10 days showed a significant tumor regression compared to control (*p* < 0.01) (Fig. 1A, B). Both treatments were well tolerated by the animals, without causing weight loss or other side effects in the mice behavior (Fig. 1C).

### Targeting Hedgehog Pathway Decreases HH Activity and Proliferation in the Xenograft Model of Uterine LMS

**Fig. 2:**
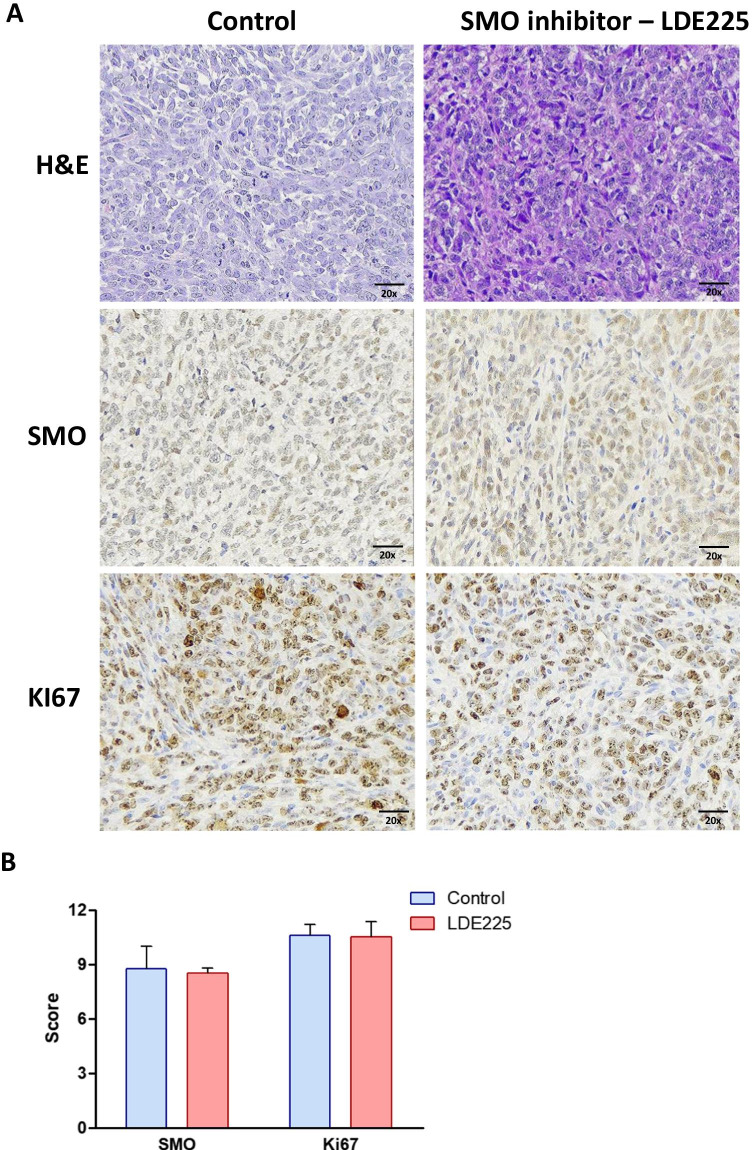
H&E and immunohistochemistry staining of SK-UT1 xenograft tumors. **A** Immunohistochemical staining for SMO and Ki67 and hematoxylin and eosin (H&E) staining of tumors treated with SMO inhibitor (LDE225) for 21 days. **B** Graph summarizing the immunohistochemical staining score (intensity x frequency) for SMO and Ki67

**Fig. 3:**
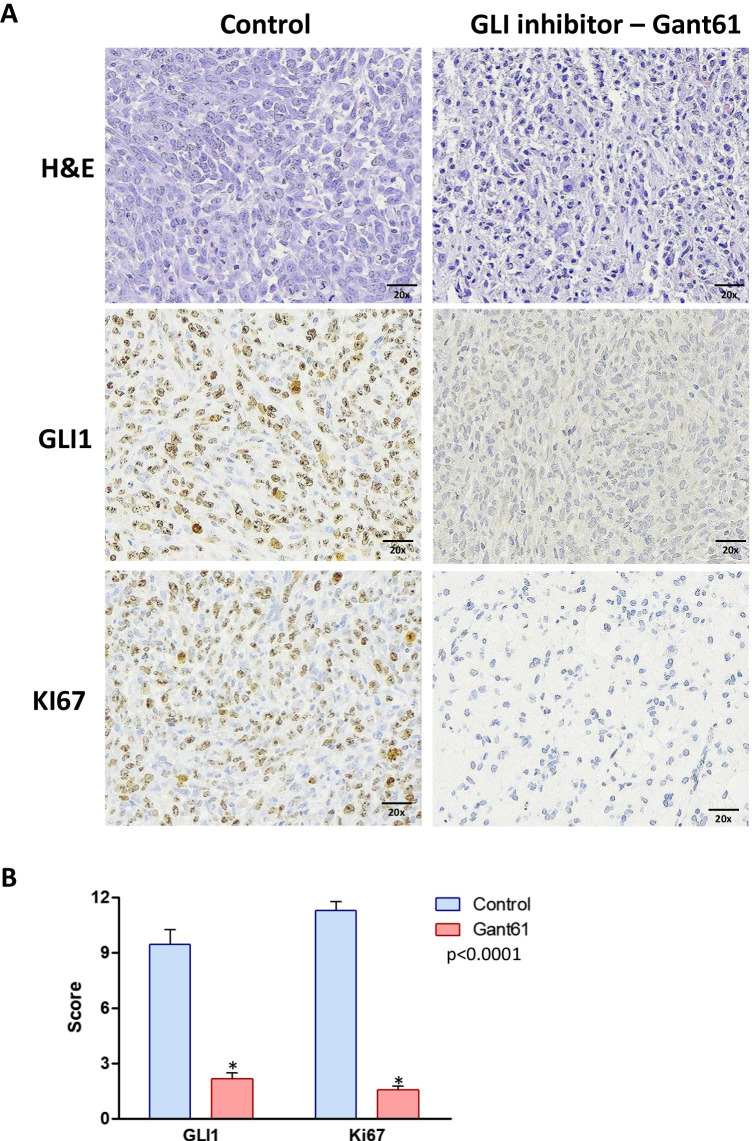
Histology and immunohistochemical analysis of the SK-UT1 xenograft tumors treated with GLI inhibitor. **A** Immunohistochemical staining for GLI1 and Ki67 and hematoxylin and eosin (H&E) staining of tumors treated with GLI inhibitor (Gant61) for 10 days. **B** Graph summarizing the immunohistochemical staining score (intensity × frequency) for GLI1 and Ki67. *p* < 0.0001

After the treatment with the HH inhibitors, LDE225 and Gant61, the tumors were collected, and H&E and IHC analyses were performed to evaluate the expression of proliferation marker (Ki67) and HH components (SMO or GLI1). The tumors treated with LDE225, SMO inhibitor, exhibited no change in the expression of SMO and Ki67 compared to the controls (Fig. 2A, B). However, the tumors treated with GLI inhibitor (Gant61) showed a significant decrease in GLI1 and Ki67 protein expression compared to the control (*p* < 0.0001) (Fig. 3A, B).

### Targeting Hedgehog Pathway Decreases Gene Expression of HH Components in Xenograft Model of Uterine LMS

**Fig. 4:**
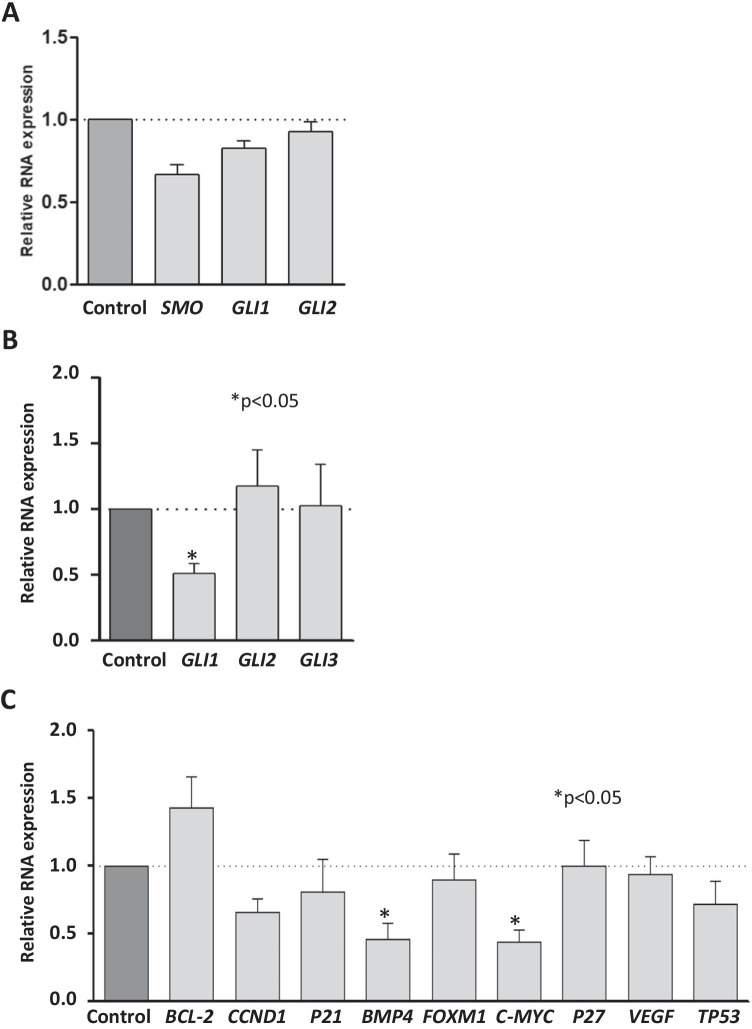
Gene expression analysis of LMS tumors treated with SMO or GLI inhibitors.** A** Relative quantification of the expression of *SMO*, *GLI1*, and *GLI2* in the LMS tumors after 21 days of treatment with LDE225 or vehicle (corn oil). **B** Relative quantification of the *GLI1*, *GLI2*, and *GLI3* in the LMS tumors after 10 days of treatment with Gant 61 or vehicle (corn oil). **C** RNA expression of GLI-target genes (*BCL-2*, *CCND1*, *P21*, *BMP4*, *FOXM1*, *C-MYC*, *P27*, *VEGF*, and *TP53*) in the LMS tumors after 10 days of treatment with Gant61 or vehicle (corn oil). *p* < 0.05

After 21 days of the treatment with SMO inhibitor (LDE225), the tumors did not show a difference in the gene expression profile of *SMO*, *GLI1*, or *GLI2* when compared to the control group (Fig. 4A). The tumors treated for 10 days with GLI inhibitor (Gant61) showed a decreased expression of *GLI1* compared to control (*p* < 0.05). Gant61 treatment did not alter the expression of *GLI2* and *GLI3* in LMS tumors (Fig. 4B). GLI-targeted genes (*BCL-2*, *CCND1*, *P21*, *BMP4*, *FOXM1*, *C-MYC*, *P27*, *VEGF*, and *TP53*) were selected to assess their expression after the Gant61 treatment. The expression of *BMP4* and *c-MYC* was significantly decreased in the Gant61-treated tumors compared to the control group (*p* < 0.05). The expression of other GLI-targeted genes (*BCL-2*, *CCND1*, *P21*, *FOXM1*, *VEGF*, and *TP53*) did not show a significant difference after treatment with Gant61 (Fig. 4C).

## Discussion

To the best of our knowledge, this study was the first to assess the effect of the SMO and GLI inhibitors on LMS growth in the xenograft model and determined if both inhibitors were capable of suppressing the activation of the HH signaling pathway in LMS in vivo. We have demonstrated previously that the HH signaling pathway is deregulated in LMS [25, 26]. Our key finding was the upregulation of SMO and the transcription factor GLI1 in LMS cell line compared to normal uterine myometrium cells [25] and human samples [26]. Moreover, inhibition of the HH pathway using SMO and GLI inhibitors suppressed the LMS proliferation and migration and increased LMS apoptosis rate in vitro.

Based on our previous results, the use of the LMS xenograft model to confirm the efficacy of SMO and GLI inhibitors was needed to understand the deregulation of HH pathway in LMS, as well as therapeutic options for this aggressive cancer. In this study, the treatment with LDE225, the SMO inhibitor, showed an inefficient suppression of LMS tumor growth, concomitantly with no changes in SMO and Ki67 protein expression, as well as in gene expression of *SMO*, *GLI1*, and *GLI2*.

Several studies demonstrated that LDE225 as an SMO inhibitor exhibited an anti-cancer efficacy in different types of tumors via blocking the HH pathway activity. In an animal model with glioblastoma, the treatment with LDE225 decreased the tumor size with downregulation of GLI1, GLI2, PTCH1, and SMO [50]. In a pancreatic tumor, LDE225 blocked the activation of the HH signaling pathway, decreasing GLI1 and PTCH1 gene expression (51]. In melanoma, LDE225 decreases the tumor size with a decrease of GLI1 expression [52].

Our results showed that the administration of SMO inhibitor (LDE225) in the animal model of LMS was inefficient to suppress the tumor growth. This result is inconsistent with our previous report showing that LDE225 treatment revealed an efficiency to block the HH pathway activity, inducing a significant decrease in cell migration, proliferation, and increased apoptosis rate in LMS cells in vitro [26].

Studies comparing in vitro and in vivo experiments have shown to be complex due to the difficulty of reproducing the tumor and non-tumor cell interactions [53]. Cancer treatment has been challenging because of the complexity and heterogeneity of the tumor [54, 55]. Tumor progression is profoundly influenced by the interactions of cancer cells with their environment. The tumor microenvironment consists of different non-cancer cell types and their stroma, which have a role in the structure, physiology, and function of the tumor [56]. In vitro experiments have been helpful to understand cell behavior, but they have cell bioactivities different from the in vivo response. The animal models mimic these complex interactions with their surrounding cells [53].

Gant61 as a GLI inhibitor has been used as a novel anti-cancer drug in preclinical studies [57]. In our previous studies, we demonstrated that GLI1 exhibited a higher expression in LMS compared to the myometrium and benign leiomyoma [25]. We also showed that the treatment with Gant61 blocked the GLI1 expression, decreasing cell proliferation and migration and increasing apoptosis index in LMS cells [26]. In this study, we tested the efficiency of Gant61 used as a therapeutic option to treat uterine LMS in the xenograft model, and our results showed a remarkable regression of the LMS growth with decreased expression of Gli and GLI-target genes *BMP4* and *c-MYC*. The use of Gant61 as a treatment option to LMS may have a potential promising outcome compared to other agents to overcome drug resistance. Recently, Nakae et al. [46] showed that CD70 antibody conjugate was able to inhibit the LMS tumor growth in a PDX model. This model possesses several advantages including preserving tumor heterogeneity and lineage hierarchy, allowing for effective chronological tumor size monitoring, and potential applications in personalized medical treatments. In this regard, evaluating of Gli inhibitor in the PDX model is warranted.

The use of Gant61 to block the HH pathway has been described in other types of tumor. For instance, in breast cancer, Gant61 decreases tumor growth [20]. In thyroid tumors, the treatment downregulated the GLI1 protein expression and reduced the tumor volume [48]. In a xenograft model of osteosarcoma, Gant61 promoted tumor regression (49). *c-MYC* and *BMP4* have been described as GLI target genes [58, 59]. In medulloblastoma, treatment with SMO inhibitor, GDC0449, showed a decrease in the c-MYC RNA expression and protein levels [59]. We previously described the upregulation of BMP4 in FFPE samples in LMS [25], and in human colon carcinoma, BMP4 expression was increased after stimulation with HH agonists [58].

In conclusion, our studies demonstrated for the first time, to the best of our knowledge, that GLI inhibitor (Gant61), but not SMO inhibitor (LDE225), showed a potent inhibitory effect on LMS tumor growth and concomitantly suppressed the expression of GLI1 and GLI-targeted genes in the xenograft model of uterine LMS. Our studies suggest that Gant61 targeting HH pathway might be considered a promising therapeutic option to inhibit the LMS progression.

## Data Availability

All data generated or analyzed during this study are included in this published article.
